# Qishen Yiqi Drop Pill improves cardiac function after myocardial ischemia

**DOI:** 10.1038/srep24383

**Published:** 2016-04-14

**Authors:** Chen JianXin, Xu Xue, Li ZhongFeng, Gao Kuo, Zhang FeiLong, Li ZhiHong, Wang Xian, Shang HongCai

**Affiliations:** 1 Beijing University of Chinese Medicine, Beijing 100029, China; 2 School of Electronic and Information Engineering, South China University of Technology, Guangzhou 510641, China; 3 Department of Chemistry, Capital Normal University, Beijing 100048, China; 4 College of Life Science, Northwest A&F University, Yangling, Shannxi 712100, China; 5 First Clinical Medical College, Dongzhimen Hospital, Beijing University of Chinese Medicine, Beijing 100700, China; 6 Key Laboratory of Chinese Internal Medicine of Ministry of Education and Beijing, Dongzhimen Hospital, Beijing University of Chinese Medicine, Beijing 100700, China

## Abstract

Myocardial ischemia (MI) is one of the leading causes of death, while Qishen Yiqi Drop Pill (QYDP) is a representative traditional Chinese medicine to treat this disease. Unveiling the pharmacological mechanism of QYDP will provide a great opportunity to promote the development of novel drugs to treat MI. 64 male Sprague-Dawley (SD) rats were divided into four groups: MI model group, sham operation group, QYDP treatment group and Fosinopril treatment group. Echocardiography results showed that QYDP exhibited significantly larger LV end-diastolic dimension (LVEDd) and LV end-systolic dimension (LVEDs), compared with the MI model group, indicating the improved cardiac function by QYDP. ^1^H-NMR based metabonomics further identify 9 significantly changed metabolites in the QYDP treatment group, and the QYDP-related proteins based on the protein-metabolite interaction networks and the corresponding pathways were explored, involving the pyruvate metabolism pathway, the retinol metabolism pathway, the tyrosine metabolism pathway and the purine metabolism pathway, suggesting that QYDP was closely associated with blood circulation. ELISA tests were further employed to identify NO synthase (iNOS) and cathepsin K (CTSK) in the networks. For the first time, our work combined experimental and computational methods to study the mechanism of the formula of traditional Chinese medicine.

Myocardial ischemia (MI) is an important type of coronary heart disease, which is characterized by ischemia in the heart muscle. Despite the advances in drug development, MI is still a leading cause of death globally. Treatment for myocardial ischemia is directed at improving blood flow to the heart muscle and may include medications, a procedure to open blocked arteries or coronary artery bypass surgery^1^,^2^,^3^,^4^,^5^. Traditional Chinese medicine (TCM) has fought against MI and its related diseases for more than 1000 years and has accumulated thousands of herbal formulas as well as clinical studies^6^,^7^.

As a well-known TCM for treating myocardial ischemia, Qishen Yiqi Drop Pill (QYDP) comprises four herbal medicines: Radix Astragali Mongolici, salvia miltiorrhiza bunge, Panax notoginseng and dalbergia odorifera. Recent research has found that QYDP can act on MI by different targets of renin-angiotensin-aldosterone system (RAAS), especially renin and Ang II, ACE/ACE2, and AT1/AT2, which eventually decrease the levels of the matrix metalloproteinase-9 and transforming growth factor-β^8^. However, the pharmacological mechanism of Qishen Yiqi Drop Pill is still uncomprehensive and unclear.

Metabonomics, a science handling with small molecule metabolites, addresses to a combination of data-rich analytical methods combined with chemometrics for profiling metabolism and explaining metabolic fingerprints in complex biological systems^9^,^10^,^11^. It has been widely used to assess organism response to disease processes, physiological stressors, or drug therapy and toxicity^12^,^13^,^14^,^15^,^16^. Nuclear magnetic resonance (NMR) spectroscopy, with the advantages of rapid, non-destructive and high-throughput, has been widely used in metabonomic research^17^,^18^,^19^,^20^,^21^. Previous works have indicated that NMR- based metabonomics analysis is an effective approach for assessing the therapeutic effects of TCMs, also for identifying metabolites with significantly changed levels and metabolic pathways intervened in TCM treatment^22^,^23^,^24^.

In our previous clinical research, the effectiveness and safety of QSYQ for the secondary prevention of MI was identified based on a randomized clinical trial composed of 3505 patients^25^, and in this research, in order to unveil the deeper detailed mechanism of QYDP, we first divided 64 male Sprague-Dawley (SD) rats into four groups: MI model group, sham operation group, QYDP treatment group and Fosinopril (a representative drug for the treatment of high blood pressure) treatment group. Echocardiography tests were employed to evaluate the therapeutic effect of QYDP, and ^1^H-NMR profiling for the four groups were studied, in order to identify the significantly changed metabolites in the QYDP treatment group. Then, the protein-metabolite interaction networks were constructed to find out the QYDP-related proteins. QYDP-related pathways were further identified, and ELISA tests were used to validate the networks, which helped to unveil the pharmacological mechanism of QYDP.

## Results

### Therapeutic effect of Qishen Yiqi Drop Pill (QYDP) analyzed by echocardiography

**Figure 1:**
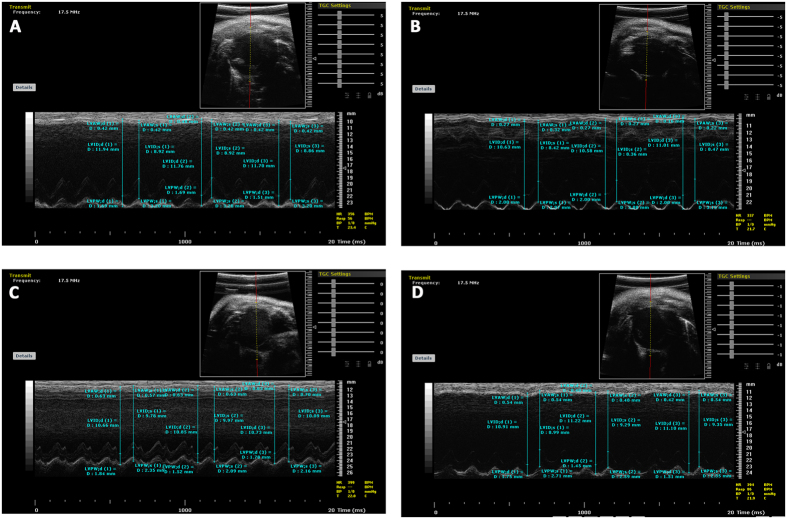
Representative M-mode echocardiographic view showing measurement of left ventricular internal dimensions for calculation of fractional shortening from the short-axis view of the left ventricle in the myocardial ischemia (MI) model group (**A**), sham operation group (**B**), Qishen Yiqi Drop Pill (QYDP) treatment group (**C**) and Fosinopril treatment group (**D**), respectively. IVS: Interventricular septum. LVPW: Left ventricular posterior wall. LVIDd: Left ventricular diastolic internal dimension. LVIDs: Left ventricular systolic internal dimension.

In order to validate the therapeutic effect of QYDP, echocardiographic parameters of left ventricular systolic (LV) function, i.e. LV end-diastolic dimension (LVEDd), LV end-systolic dimension (LVEDs), Fractional shortening (FS) and ejection fraction (EF) were measured for the 64 rats by M-mode echocardiogram (Fig. 1). EF was expressed as the ratio of the left ventricular stroke volume (SV) to the left ventricular end-diastolic volume (LVEDV), and FS was obtained by measuring the LVEDd and LVEDs, dividing the difference by the LVEDd. Compared with sham operation group, EF and FS in the MI group showed significant decline (P = 0.003 and 0.003 for LVEDd and LVEDs, respectively). Since EF and FS were taken as measures of systolic function, the declined EF and FS showed impaired systolic function in the MI group, indicating the reliability of the experiment. In addition, the left ventricular internal diameter in diastole (LVEDd) and systole (LVEDs) were much higher in rates with the QYDP treatment group than the MI model group (772.5 ± 98.34 μl vs 699.3 ± 102.09 μl for LVEDd; and 644.49 ± 111.02 μl vs. 459.10 ± 109.28 μl for LVEDs), showing the improvement of cardiac function by QYDP. Furthermore, compared with the Fosinopril treatment group, LVEDd and LVEDs were significantly higher in the QYDP treatment group, but no significant change could be found in EF and FS (34.52 ± 9.73% vs. 32.31 ± 10.07% for EF and 17.88 ± 3.55% vs. 19.38 ± 3.63% for FS). The result showed QYDP might have greater effect on the treatment of MI, and presented similar systolic function in rats as Fosinopril. This result was in agreement with previous study, which showed the effectiveness and safety of QYDP for the secondary prevention of MI^25^,^26^,^27^.

### ^1^H-NMR analysis of plasma samples

**Figure 2:**
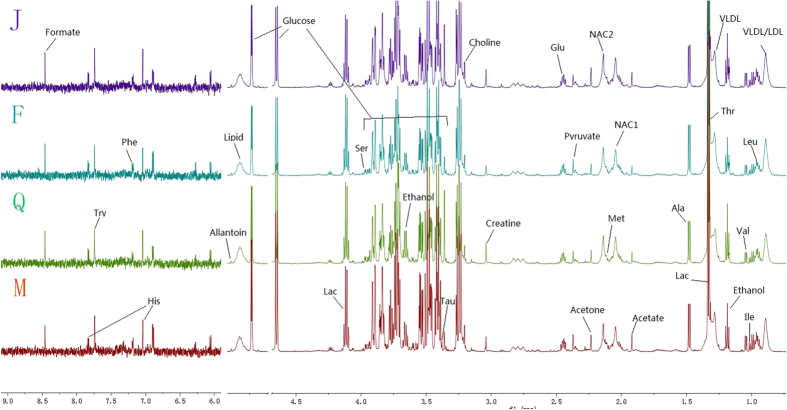
600 MHz representative ^1^H NMR (δ9.5–5.20, 4.70–0.50) CPMG spectra of serum samples of MI model group (**M**), sham-operation group (**J**), QYDP treatment group (**Q**) and Fosinopril treatment group (**F**).

Plasma contains almost all of the low molecular weight species in whole blood and a few high molecular weight compounds, and NMR spectra of plasma under similar physiological conditions are highly reproducible, thus making them useful for the diagnosis of metabolic and diseased states. Figure 2 shows representative 600 MHz ^1^H NMR CPMG spectra of plasma from MI model group, sham operation group, Fosinopril treatment group and QYDP treatment group. The plasma NMR spectra were dominated by LDL/VLDL (δ0.86, δ1.26), leucine (δ0.95, δ0.97), valine (δ1.03), lactate (δ1.33, δ4.12), alanine (δ1.48), acetate(δ1.92), glutamate(δ2.14), pyruvate (δ2.38), glucose(δ3.2–4.0, 4.66, 5.23) ect^23^,^24^,^28^. To obtain detailed analysis of metabolic differences among the groups, multivariate data analyses including PCA and OPLS-DA were performed.

**Figure 3:**
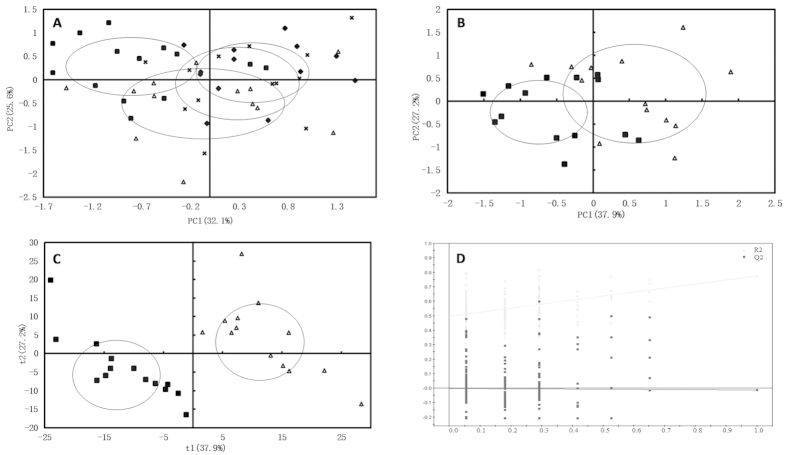
(**A**) PCA score plot of all samples from sham operation group, MI group, Fosinopril treatment group and QYDP treatment group; (**B**) PCA score plot of MI model group and Sham operation group; (**C**) PLS-DA score plot of MI model group and Sham operation group; (**D**) The validation plot derived from MI model group and sham operation group. Solid square for the rats in sham operation group, hollow triangle for the MI group, solid diamond for the Fosinopril treatment group, and cross for the QYDP treatment group.

There are three fundamental approaches to the problem of outlier detection, including unsupervised clustering, supervised classification and semi-supervised recognition. In this work, the unsupervised classifier PCA was selected and employed to identify the outliers in the 64 subjects (Fig. 3A), and the result showed no outlier in 95% confidence limit. Furthermore, PCA was performed for the MI group and the sham-operation group (Fig. 3B), and small overlap (R^2^X = 0.651, Q^2^Y = 0.321) between the two groups indicated the success in constructing the MI rat model. PLS-DA was further performed to enhance this separation. The score plot (Fig. 3C) showed a satisfactory discrimination between the two groups (R^2^X = 0.225, R^2^Y = 0.912, Q^2^Y = 0.603). The validation plot (Fig. 3D) showed high R^2^X and Q^2^Y values, and thus illustrated that the model was robust.

**Figure 4:**
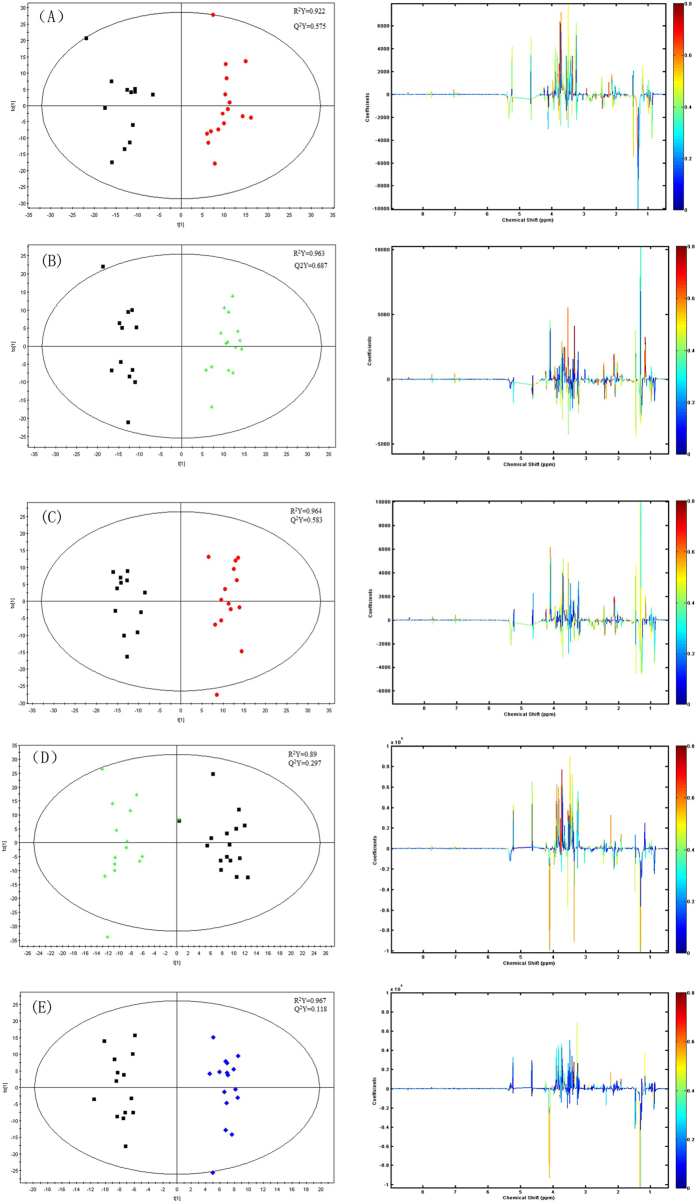
OPLS-DA score plots (left) and corresponding color-coded correlation coefficient loading plots (right), (**A**) MI model group vs Sham operation group, (**B**) MI model group vs QYDP treatment group, (**C**) MI model group vs Fosinopril treatment group, (**D**) QYDP treatment group vs Sham operation group, (**E**) Fosinopril treatment group vs Sham operation group.

To identify the significantly changed metabolites among the groups, pairwise OPLS-DA models were built. Figure 4 revealed the OPLS-DA score plots for pairewise comparison of MI model group, sham-operation group, QYDP treatment group and Fosinopril treatment group, along with the corresponding coefficients plots depicting the major discriminators. Compared with MI model group, the administration of QYDP led to elevation of acetone, formate and methionine, along with allantoin, leucine, alanine, valine, isoleucine, glutamine, theronine and pyruvate. In the F and the QYDP groups, threonine showed great higher level compared with the MI group. Table 1 showed the biomarkers of QYDP in plasma.

### Metabolites identification of ^1^H NMR spectra of cardiac muscle

**Figure 5:**
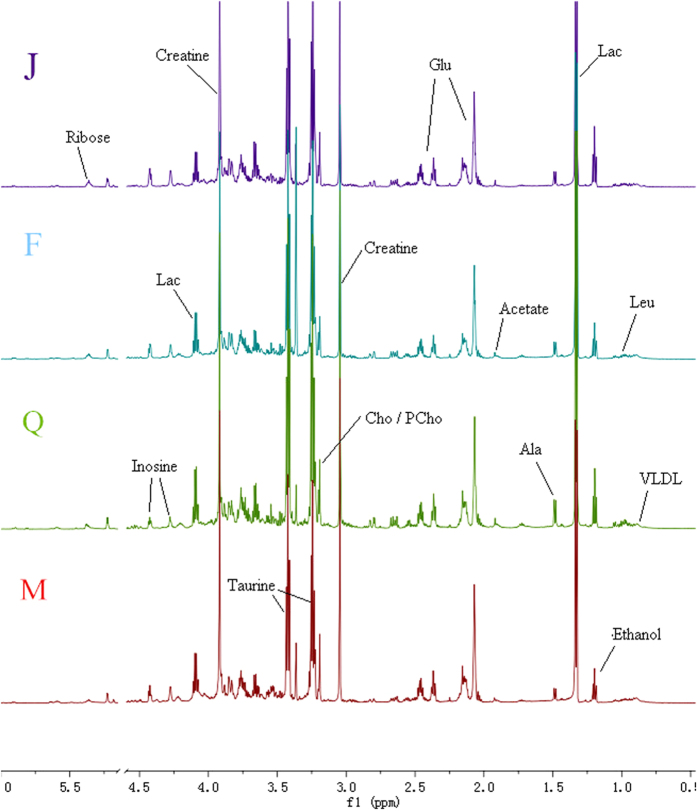
600 MHz representative ^1^H NMR (δ9.5–5.20, 4.70–0.5) of cardiac muscle samples of MI model group (**M**), sham-operation group (**J**), QYDP treatment group (**Q**) and Fosinopril treatment group (**F**).

Figure 5 shows representative 600 MHz ^1^H NMR NOESY spectra of cardiac muscle from model group, sham operation group, Fosinopril treatment group and QYDP treatment group. The cardiac muscle NMR spectra were dominated by valine (δ0.99), lactate (δ1.33, δ4.12), acetate(δ1.92), glutamate(δ2.36), pyruvate (δ2.38), glutathione (δ2.52), methionine (δ2.655), creatine (δ3.05), choline (δ3.19), taurine (δ3.41), inosine (δ4.26), ribose (δ5.36), glucose(δ3.2–4.0, 4.66, 5.23), and so on. In the ^1^H NMR spectra of cardiac muscle samples, the dominate change of the signals from low molecular weight metabolites such as lactate, methionine, creatine, choline and taurine were detected. These results showed the reliability of our NMR experiments. In the following two paragraphs, we pointed out the significantly changed metabolites in the QYDP treatment group, compared with the MI model group.

**Figure 6:**
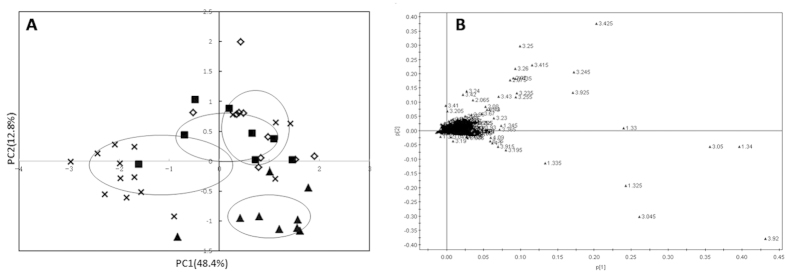
PCA results of cardiac muscle ^1^H-NMR spectra. (**A**) A score plot of all samples from sham operation group, MI group, Fosinopril treatment group and QYDP treatment group. (**B**) A loading plot of all samples from the four groups. Solid square for the rats in sham operation group, solid triangle for the MI group, hollow diamond for the Fosinopril treatment group, and cross for the QYDP treatment group.

The PCA score plot showed clear-cut separation between the model group (solid triangle) and the other three groups along PC1 (Fig. 6), indicating that the model group was successfully set up. Furthermore, the QYDP treatment group and Fosinopril treatment group are not separated from the sham-operation group along PC1, but divided slightly along PC2, suggested that the metabolic profiles of these three groups were similar and the metabolic profiles of medicine administration groups were not return to normal completely.

Table 2 showed the variation of the integrals of the normalized spectral regions responsible for different serum metabolites and lists the results from the student’s t-test (p < 0.05) for comparison. Compared to MI model group, the levels of taurine, aspartate, valine, ribose, acetate, methionine and glucose were increased while levels of inosine, creatine, choline, glutamate, pyruvate, lactate and glutathione were decreased in the QYDP treatment group. In addition, the F and the QYDP groups showed significantly increased level of taurine compared with that of MI group, while presented much lower levels of creatine and choline.

### Metabolite-protein networks and related protein pathways

**Figure 7:**
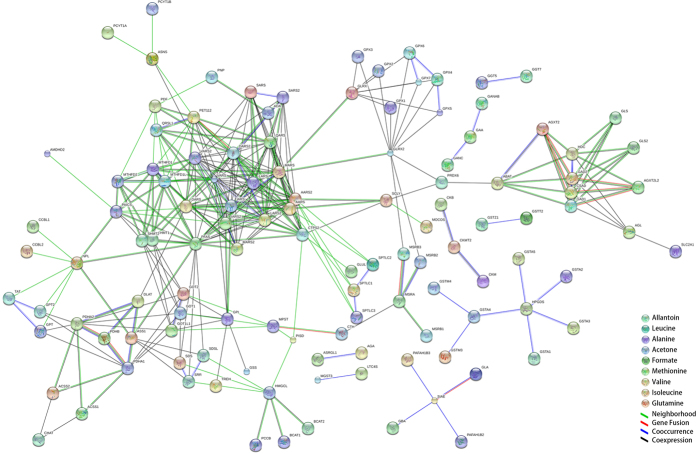
9 metabolite biomarkers for QYDP associated with 199 proteins. Metabolites include Allantoin, Leucine, Alanine, Acetone, Formate, Methionine, Valine, Isoleucine, and Glutamine.

In order to identify the metabolite-related proteins, we first collected 9 representative metabolite biomarkers derived from the plasma- and the cardiac muscle-based ^1^H NMR profiling (Allantoin, Leucine, Alanine, Acetone, Formate, Methionine, Valine, Isoleucine, and Glutamine), and constructed the metabolite-protein networks for QYDP. The result showed that 199 proteins were identified for the QYDP-related metabolites (Fig. 7).

**Figure 8:**
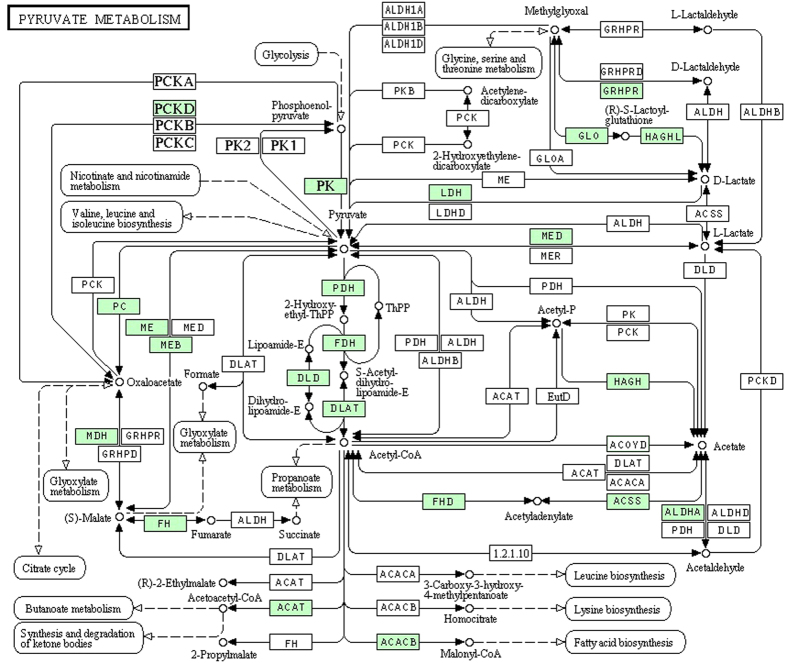
Pyruvate metabolism pathway derived from the identified 20 QYDP-related proteins on the metabolite-protein networks. The 20 proteins are underlined with green color.

We further projected the 199 QYDP-related proteins to the protein pathways (supplemental information Table S1). Pyruvate metabolism was reported to regulate blood glucose^29^, showing the relationship between 29 protein-related pyruvate metabolism pathway and blood glucose (Fig. 8). In addition, retinol was the predominant circulating form of vitamin A in the blood^28^, suggesting the importance of 18 protein related-retinol metabolism pathway in blood. Moreover, tyrosine was found to be able to reduce blood pressure^30^, showing the association between 17 protein-related tyrosine metabolism pathway and blood flow. Previous study also reported the association of the purine metabolism with primitive erythrocytes, indicating the relationship between the 26 protein-related purine metabolism and the blood circulation. These results indicated the associations of pyruvate metabolism pathway, retinol metabolism pathway, tyrosine metabolism pathway and purine metabolism pathway with blood circulation, suggesting that it was mainly the 4 identified pathways that QYDP improved the cardiac function, and thus played a therapeutic role for MI.

### ELISA analysis

To validate the QYDP-related proteins in section 3.5, 3 proteins (endothelial NO synthase (eNOS), inducible NO synthase (iNOS), and cathepsin K (CTSK)) were selected from the metabolite-protein networks, and their protein levels were determined by ElISA. The eNOS level in the QYDP treatment group was significantly higher than that in the MI model group (17.87 ± 2.04 ng/ml vs. 14.93 ± 1.04 ng/ml), while no significant change in the iNOS level was found in the M and the QYDP treatment groups. For CTSK, the protein level in the QYDP treatment group also exhibited significant change, compared to the MI model group (951.88 ± 544.53 ng/ml vs. 2020.64 ± 960.68 ng/m). The results showed that the biomarker proteins in QYDP included eNOS and CTSK, which indicated the reliability of the metabolite-protein networks.

## Discussion

In our previous work, we tested the effectiveness and safety of QSYQ for the secondary prevention of MI based on a randomized clinical trial composed of 3505 patients, and the result showed that QSYQ had similar effects to aspirin in the secondary prevention of MI^31^. To further explore the mechanism of QSYQ, we collected the plasma and cardiac muscle samples of 64 rats, and identified the significantly changed metabolites in the QSYP treatment group by metabolomics analysis in this work. The results showed that Allantoin, Leucine, Alanine, Acetone, Formate, Methionine, Valine, Isoleucine, and Glutamine were representative metabolites after QYDP treatment. Valine, Isoleucine and Leucine are branched chain essential amino acids (BCAA), which are critical to human life and are particularly involved in stress, energy and muscle metabolism. BCAA are particularly responsive to the inhibitory insulin action on amino acid release by skeletal muscle and their metabolism is profoundly altered in conditions featuring insulin resistance, insulin deficiency, or both^32^. Especially, Leucine stimulates insulin release, which in turn stimulates protein synthesis and inhibits protein breakdown^33^. In addition, Alanine is highly concentrated in muscle and is one of the most important amino acids released by muscle, functioning as a major energy source. It is an important participant as well as regulator in glucose metabolism, and its levels always parallel blood sugar levels^34^. Since the levels of leucine, isoleucine, valine and alanine were higher than normal in the model group, but decreased obviously in the QYDP treatment group, our work indicated that the QYDP could significantly regulate the amino acids metabolism by reducing the content of leucine, isoleucine, valine and alanine.

Moreover, the decreased level of choline after the QYDP treatment in the cardiac muscle tissue shows good agreement with the heart-specific overexpression of choline acetyltransferase that catalyzed the acetylation of choline and protected heart against ischemia^35^. This also implies the cardiac muscule-specific of choline. In addition, the elevation of methionine level in the plasma samples of the QYDP group is supported by previous work, which showed that methionine had beneficial effects on the treatment of cardiovascular lesions^36^. Also, the increased level of taurine in the cardiac muscle tissue after the QYDP and the Fosinopril treatment shows good agreement with the result of previous study that indicated taurine could be of benefit in cardiovascular disease of different etiologies^37^.

To further explore the mechanism of QYDP, we constructed the metabolite-related networks, and identify 81 QYDP-related proteins and 4 QYDP-related signaling pathways, including pyruvate metabolism pathway, retinol metabolism pathway, tyrosine metabolism pathway and purine metabolism pathway. Obviously, the 4 explored pathways presented good consistency with the analyzed results for the metabolites in the QYDP treatment group. In addition, stimulation of pyruvate metabolism was reported to lead to activation of adenosine triphosphate (ATP)–sensitive potassium channels, thus regulating the blood gulcose^29^. Retinol was found to be the predominant circulating form of vitamin A in the blood^38^. Tyrosine makes from another amino acid called phenylalanine, which is an essential component for the production of several important brain chemicals. Low levels of blood tyrosine have been associated with low blood pressure^39^.

Therefore, the 4 pathways were closely related to blood circulation, which validated the therapeutic effect of QYDP for MI. The ELISA further examined the concentrations of 3 QYDP-related proteins in the QYDP group compared with the MI model group, showing the levels of 2 proteins were significantly higher in the former. These results suggest that the 81 identified QYDP-related proteins configured a target scope for QYDP, and thus provided an efficient clue to further study the targets of QYDP in the future work.

## Conclusion

In the present study, echocardiography analysis showed the therapeutic effects of QYDP in the treatment of MI. ^1^H NMR-based metabonomics method combined with multivariate data analysis was employed to study the metabolic mechanism of QYDP. The QYDP could regulate the metabolic disorders and promote the regression of metabolic phenotype close to the normal range by adjusting the levels of certain metabolites in the amino acid metabolism. Based on the metabolite biomarkers, the QYDP-related proteins were identified, and the related pathways were found. The pathways showed QYUP was closely associated with blood circulation. ELISA tests further validated the biomarker proteins of QYDP. Our work unveiled that QYDP could improve the cardiac function after MI, mainly by the 81identified QYDP-related proteins and the 4 QYDP-related pathways, which would help to speed up the clinical application process of the formula.

## Materials and Methods

### Materials

QYDP was purchased from Tianjin Tasly Pharmaceutical Co., Ltd. (Tianjin, China). Fosinopril was purchased from Bristol-Myers Squibb Co., Ltd. (Shanghai, China). D_2_O (99.9%) was purchased from Cambridge Isotope Laboratories. CH_3_OH (HPLC Grade) was purchased from Thermo Fisher scientific (Shanghai, China). CH_3_Cl (AR) was purchased from Beijing Chemical Works (Beijing, China). eNOS Elisa Kit (No:ab166866) was purchased from Abcam Co., Ltd. (Shanghai, China). iNOS Elisa Kit (No:CSB-E08148h ) was purchased from Cusabio Co., Ltd. (Wuhan, China). CTSK Elisa Kit (No:SEA267Ra ) was purchased from USCNK Co., Ltd. (Wuhan, China).

### Animals and drug administration

All animal experimental methods were carried out in accordance to the guidelines of China legislations on the ethical use and care of laboratory animals. And all experimental protocols were approved by the Ethics Committee in Beijing University of Chinese Medicine. In this work, 64 male Sprague-Dawley(SD) rats (10–12 weeks old, weighing 240 ± 10 g at the start of the experiment) were purchased from Charles River Laboratories in China. The rats were housed one per cage and with free access to food and water under a 12 h/12 h light/dark cycle at 23 ± 2 °C. To construct the rat models of Myocardial Ischemia, 48 rats were first randomly selected to be anesthetized with sodium pentobarbital (60 mg/kg IP), and Myocardial ischemia was induced by ligating the left anterior descending (LAD) coronary artery^28^. Electrocardiograms (ECG) were monitored by FX 7200 elctrocardiograph (Fukuda Denshi Co., Ltd., Tokyo, Japan) to confirm that the MI model was set up successfully. Other 16 rats were selected to be anesthetized with sodium pentobarbital, opened the thorax with no ligating the left anterior descending (LAD) coronary artery, which were considered as the sham group. Finally, all the rats were divided into 4 groups undergoing (1) sham operation (Sham group, n = 16), (2) MI modelling (MI group, n = 16), (3) QYDP treatment (QYDP group, n = 16), and (4) Fosinopril treatment (Fosinopril group, n = 16), respectively. In the Sham (n = 16) and MI (n = 16) groups, rats received saline. Meanwhile, rats in the QYDP treatment group and Fosinopril treatment group were daily given QYDP (0.15 g/kg) and Fosinopril (1.2 mg/kg) respectively, which derived from the equivalent conversion between animals and people by body surface area based on the recommended daily human dosage (7.5 g/kg for QYDP and 4.8 mg/kg for Fosinopril)^25^. They were fed in metabolism cages which were in an environmentally controlled breeding room during the whole period, with standard laboratory food and water. The experiments lasted for 48 days.

### Echocardiography

The study was conducted in Department of Echocardiography, Beijing An Zhen Hospital, Capital Medical University. Each rat was anesthetized with 60 mg/kg sodium pentobarbital, with the left side of the chest shaved to gain a clear image. The 64 rats were studied in the left lateral decubitus position for serial echocardiographic examinations. High-resolution *in vivo* imaging system (VisualSonics Vevo 2100) was employed for Doppler echocardiography. Transmitral flow velocity profile was determined by positioning a sample volume at the tip of the mitral valve on the para-apical long-axis view. The Doppler beam was set with <30° of the incident angle to flow direction identified on color Doppler image. The peak velocity and deceleration time of the early diastolic filling wave were measured. The deceleration time was obtained by extrapolating the initial slope of early diastolic filling wave deceleration to the baseline. Transmitral inflow pattern was recorded on a strip chart at 100-mm/s sweep speed with simultaneous 3-lead ECG for offline analysis. All measurements represent the mean of 5 consecutive cardiac cycles, and heart rate was calculated on the basis of the strip chart of Doppler echocardiography.

### Sample collection and preparation

After consecutive administration for 48 days, all the rats were sacrificed for plasma and cardiac muscle samples. Plasma and cardiac muscle samples were snap frozen in liquid nitrogen, and stored at −80 °C until metabolomics analysis.

Plasma samples were thawed in a fume hood, and were prepared by mixing 200 μl of serum with 400 μl of 1.5 M of deuterated phosphate buffer (NaH_2_PO_4_ and K_2_HPO_4_, including 0.1% TSP, pH 7.47), adding D_2_O up to 600 μl if the volume of serum is insufficient. The mixture was left to stand for 5 min at room temperature and then centrifuged at 13000 rpm at 4 °C for 15 min. The supernatant solution (550 μl) was then transferred into a 5 mm NMR tube for NMR analysis.

Cardiac muscle samples(about 110 mg) were thawed at room temperate, and add 4 mL/g (wet weight) of 100% methanol and 1.25 mL/g deionized water (wet weight), prepared for homogenate. Add 4 mL/g (wet weight) of 100% CHCl_3_ and 1.25 mL/g deionized water (wet weight) into the homogenate to extract. After a 10 min incubation at 4 °C, the extract samples were centrifuged at 5000 g for 10 min at 4 °C. The upper aqueous phase was lyophilized. The powder of the extract was dissolved in 600 μl of phosphate buffer(0.1 M Na_2_HPO_4_/ NaH_2_PO_4_, PH = 7.4), centrifuged at 13000 g for 15 min at 4 °C. The supernatant solution (550 μl) was then transferred into a 5 mm NMR tube for NMR analysis.

### NMR analysis

All the samples were analyzed at 298 K using a VARIAN VNMRS 600 MHz NMR SPECTROMETER operating (Varian Inc, Palo Alto, Calif) at 599.871 MHz using a 5-mm inverse-proton (HX) triple resonance probe with z-axis gradient coil.

^1^H NMR spectra of plasma were recorded using the water-suppressed standard 1D CPMG pulse sequence (RD-90°-(τ-180°-τ)n-ACQ), where a fixed total spin–spin relaxation delay 2nτ of 320 ms was applied to attenuate the broad NMR signals from slowly tumbling molecules (such as proteins) and retain those from low-molecular weight compounds and some lipid components. The free induction decays (FIDs) were collected into 64 K data points with a spectral width of 12000 Hz and 128,scans. The FIDs were zero-filled to double size and multiplied by an exponential line-broadening factor of 0.5 Hz before Fourier transformation (FT). NMR spectra of the cardiac muscle extracts samples were acquired using a standard sequence: one dimensional spectrum using the first increment of the NOESY pulse sequence (RD–90°–t_1_–90°–tm–90°–ACQ) with water suppression, the water suppression was achieved with an irradiation on the water peak during the relaxation delay (RD = 2.0 s) and a mixing time, tm, of 100 ms. t_1_ was set to 4 μs. The 90° pulse length was adjusted to approximately 9.8 μs, and 128 transients were collected into 64 K data points for each spectrum with a spectral width of 20 ppm, The FIDs were weighted by an exponential function with a 0.5 Hz line-broadening factor prior to Fourier transformation. Standard COSY, TOCSY, HMBC and J-resolved spectra were also acquired for metabolite identification purposes for the selected plasma and cardiac muscle extracts samples.

All of the ^1^H NMR spectra were manually phased, corrected for baseline distortion by MestReNova7.1.0 software (Mestrelab Research, Spain). All the spectra were referenced to the methyl group of lactate at δ 1.336. The region of δ 4.7–5.2 was deleted to eliminate the effects of water suppression. The spectral region δ 0.5–4.7 and δ 5.2–9.0 was automatically data reduced to 1700 integral segments of equal length (0.005 ppm). The area under the spectrum was then calculated for each segmented region and expressed as an integral value. The integrated data were normalized to the total sum of the spectrum before multivariate statistical analysis to give the same total integration value for each spectrum.

Subsequently, the integral values were imported into SIMCA-P + 12.0 (Umetrics, Sweden) for multivariate statistical analysis. The data were mean centered for principle component analysis (PCA) and partial least squares discriminant analysis (PLS-DA)^20^,^21^,^22^, and in order to improve the separation due to groups and minimize other biological analytical variation, sample classes were modeled using the OPLS-DA algorithm at a unit variance-scaled approach. The PCA and PLS-DA score plots were showed with the first principal component and the second principle component, while OPLS-DA were visualized with the first principle component and the orthogonal component. The model coefficients locate the NMR variables associated to specific intervention as y variables. The model coefficients were then back-calculated from the coefficients incorporating the weight of the variables in order to enhance interpretability of the model; in the coefficient plot, the intensity corresponds to the mean-centered model (variance) and the color-scale derives from the unit variance-scaled model (correlation). Thus, biochemical components responsible for the differences between samples detected in the scores plot can be extracted from the corresponding loadings with the weight of the variable contributing to the discrimination. The coefficient plots were generated with MATLAB scripts (downloaded from http://www.mathworks.com)with some in-house modifications and was color-coded with absolute value of coefficients (r).

### ELISA for protein levels

Levels of rat endothelial NO synthase (eNOS), inducible NO synthase (iNOS), and cathepsin K (CTSK) were measured in plasma using commercial ELISA kits according to the manufacturer’s instructions.

Briefly, rat cardiac muscle samples in the MI model group, sham operation group, QYDP treatment group and Fosinopril treatment group were homogenized in Tris-buffered saline (25 mM Tris-HCl, pH 7.4, 150 mM NaCl) supplemented with Protease Inhibitor Cocktail (Sigma, 250 μl per 5 ml of buffer), respectively. cardiac muscle homogenates were centrifuged at 100,000 × g for 1 h, and then diluted 1:10 before carrying out the ELISA, as detailed in the manufacturer’s protocol. Protein was quantified using the Bradford protein assay. Spectrophotometer (Bio-Tek Instruments, ICN, USA) set to a wavelength of 450 nm and corrected for absorbance at 540 nm. The final eNOS, iNOS and CTSK levels were determined following normalization to total protein levels.

### Construction of metabolite-related network

The representative metabolite biomarkers of the QYDP treatment group derived from the plasma- and the cardiac muscle-based ^1^H NMR profiling were first collected, and the corresponding proteins were then searched in the Human Metabolome Database (HMDB; http://www.hmdb.ca/), a freely available electronic database containing detailed information about small molecule metabolites found in the human body. Further, the metabolite-related proteins were mapped in the biological pathways to search the metabolite-related signal pathways in Database for Annotation, Visualization, and Integrated Discovery (DAVID; http://www.david.niaid.nih.gov). Finally, relationships among metabolite, protein and biological pathways were constructed in STRING (http://string-db.org/), a database of known and predicted protein interactions.

### Statistics

Paired t-test was used for all data analysis. Statistical significance was defined as P < 0.05.

## Additional Information

**How to cite this article**: JianXin, C. *et al.* Qishen Yiqi Drop Pill improves cardiac function after myocardial ischemia. *Sci. Rep.* **6**, 24383; doi: 10.1038/srep24383 (2016).

## Supplementary Material

Supplementary Information

## Figures and Tables

**Table 1:** Quantitative comparison of metabolites found in serum of M, J and Q group mice.

Metabolites	Interal in M group^a^(mean ± std)×10^−2^	Interal in J group^a^(mean ± std)×10^−2^	Interal in Q group^a^(mean ± std)×10^−2^	Interal in F group^a^(mean ± std)×10^−2^	r^b^ (J vs M) (|r| > = 0.55)	p^c^ (J vs M) (p < 0.05)	r^b^ (Q vs M) (|r| > = 0.55)	P^c^(Q vs M) (p < 0.05)
allantoin	1.48 ± 0.41	0.9 ± 0.21	1.12 ± 0.20	1.0 ± 0.30	−0.78	0.0004	−0.55	0.01
leucine	11.1 ± 0.80	9.8 ± 0.75	10.0 ± 0.92	9.95 ± 1.62	−0.70	0.0001	−0.57	0.003
alanine	8.63 ± 3.1	6.24 ± 0.41	6.12 ± 0.82	6.79 ± 0.94	−0.56	0.02	−0.48	0.02
acetone	13.66 ± 1.97	20.62 ± 5.27	14.38 ± 1.96	14.83 ± 2.31	0.65	0.0001	0.17	0.36
formate	1.25 ± 0.31	2.08 ± 0.68	1.81 ± 0.51	2.38 ± 0.51	0.62	0.0003	0.62	0.002
methionine	17.5 ± 2.86	21.65 ± 2.65	21.8 ± 3.23	21.73 ± 2.73	0.61	0.001	0.59	0.001
valine	3.66 ± 0.59	3.03 ± 0.40	2.98 ± 0.43	3.15 ± 0.46	−0.61	0.002	−0.60	0.002
isoleucine	3.0 ± 0.73	2.33 ± 0.35	2.17 ± 0.41	2.39 ± 0.46	−0.61	0.002	−0.56	0.003
glutamine	2.50 ± 0.5	1.97 ± 0.24	1.87 ± 0.26	1.96 ± 0.26	−0.60	0.004	−0.63	0.001
theronine	0.56 ± 9.73	41.68 ± 10.87	45.8 ± 13.25	42.91 ± 12.85	−0.58	0.001	−0.44	0.03
serine	2.73 ± 0.66	2.07 ± 0.48	2.71 ± 1.77	2.21 ± 0.56	−0.57	0.005	−0.01	0.97
lactate	155.8 ± 37.82	135.98 ± 34.6	169.98 ± 30.78	168.71 ± 34.92	−0.34	0.16	0.24	0.29
pyruvate	15.98 ± 3.38	13.11 ± 3.10	11.13 ± 3.59	15.06 ± 4.98	−0.42	0.03	−0.56	0.001
glucose	41.89 ± 3.15	46.99 ± 2.96	44.97 ± 4.80	45.27 ± 3.41	0.68	0.0002	0.39	0.06

^a^The relative integrals of metabolites were determined from 1D ^1^H NMR analysis of serum of each group mice.

^b^The values of correlation number extracted from the correlation plots of OPLS-DA models. The cutoff values are 0.55 in the correlation-loading plot of M vs J and M vs Q.

^c^The p values were obtained from student’s t-test.

**Table 2:** Quantitative comparison of metabolites found in cardiac muscle extracts of M, J, Q and F group mice.

Metabolites	Interal in M group^a^(mean ± std)×10^−2^	Interal in J group^a^(mean ± std)×10^−2^	Interal in Q group^a^(mean ± std)×10^−2^	Interal in F group^a^(mean ± std)×10^−2^	VIP^b^ (J vs M)	p^c^ (J vs M)	VIP^b^ (Q vs M)	P^c^(Q vs M)
taurine	46.58 ± 10.13	84.10 ± 25.09	68.72 ± 18.11	82.78 ± 32.14	1.76	0.19	0.60	0.58
inosine	6.16 ± 0.41	5.19 ± 1.01	4.63 ± 0.86	5.27 ± 1.32	2.47	0.98	2.14	0.0001
creatine	207.92 ± 30.31	148.04 ± 31.08	142.90 ± 0.86	158.29 ± 26.66	3.44	0.0001	1.74	0.0001
choline	20.51 ± 2.36	14.24 ± 3.19	15.70 ± 3.38	13.36 ± 3.10	3.23	0.0001	1.48	0.003
glutamate	16.07 ± 2.44	12.70 ± 1.80	11.97 ± 2.20	13.36 ± 1.36	1.85	0.62	0.69	0.02
valine	5.17 ± 1.18	5.17 ± 1.07	6.38 ± 1.0	5.35 ± 0.86	0.31	0.87	0.95	0.04
aspartate	5.80 ± 1.36	8.50 ± 0.43	8.20 ± 1.34	8.90 ± 1.49	3.35	0.002	2.49	0.0001
pyruvate	24.19 ± 3.69	28.92 ± 6.97	22.0 ± 2.89	27.81 ± 5.78	0.48	0.42	0.74	0.02
lactate	46.6 ± 11.69	35.4 ± 5.92	25.80 ± 4.96	41.90 ± 11.20	2.73	0.001	1.47	0.0003
ribose	4.72 ± 0.69	7.61 ± 0.78	6.85 ± 1.67	7.13 ± 0.87	2.62	0.001	0.65	0.03
acetate	5.50 ± 0.96	8.73 ± 1.23	8.69 ± 1.67	8.14 ± 2.48	1.32	0.57	1.57	0.05
methionine	4.10 ± 0.30	5.06 ± 0.42	5.15 ± 0.40	4.75 ± 0.83	3.06	0.005	1.88	0.0001
glucose	10.63 ± 1.41	12.90 ± 0.30	11.12 ± 1.49	13.09 ± 1.29	2.15	0.17	1.59	0.84
glutathione	4.59 ± 0.25	3.97 ± 0.30	4.47 ± 0.22	3.80 ± 0.15	2.42	0.008	0.52	0.80

^a^The relative integrals of metabolites were determined from 1D ^1^H NMR analysis of cardiac muscle extracts of each group mice

^b^The values of correlation number extracted from the correlation plots of OPLS-DA models.

^c^The p values were obtained from student’s t-test.
