# Supplementary material for: Qishen Yiqi Drop Pill improves cardiac function after myocardial ischemia
Source: Sci Rep. 2016 Apr 14;6:24383. doi: 10.1038/srep24383 (PMC4830957; doi:10.1038/srep24383)
Supplement: Supplementary Information [file srep24383-s1.doc]

**Qishen Yiqi Drop Pill improves cardiac function after myocardial ischemia**

Chen JianXin^1,6†^, Xu Xue^2†^, Li ZhongFeng^3†^, Gao Kuo^1†^, Zhang FeiLong^2,4^, Li ZhiHong^5^, Wang Xian^6*^, Shang HongCai^1,6*^

1Beijing University of Chinese Medicine, Beijing 100029, China

2School of Electronic and Information Engineering, South China University of Technology, Guangzhou 510641, China

3Department of Chemistry, Capital Normal University, Beijing 100048, China

4 College of Life Science, Northwest A&F University, Yangling, Shannxi 712100, China

5 First Clinical Medical College, Dongzhimen Hospital, Beijing University of Chinese Medicine, Beijing 100700, China

6 Key Laboratory of Chinese Internal Medicine of Ministry of Education and Beijing, Dongzhimen Hospital, Beijing University of Chinese Medicine, Beijing 100700, China.

**Table S1 Qishen Yiqi Drop Pill-related signaling pathway**

| **Pathway** | **Count** | **Genes** | **FDR** |
| --- | --- | --- | --- |
| Metabolism of xenobiotics by cytochrome P450 | 43 | CYP3A4, CYP3A5, CYP2F1, CYP3A7, CYP1B1, CYP2C19, CYP2B6, CYP2C18, CYP2S1, ADH1C, ADH5, ADH6, ADH1B, ADH7, ADH1A, GSTM5, ALDH3A1, GSTM1, CYP3A43, GSTM2, GSTM3, GSTM4, GSTK1, ALDH1A3, GSTZ1, GSTO2, GSTO1, GSTA1, GSTA2, GSTA3, GSTA4, GSTT2B, CYP1A1, GSTA5, CYP2C9, CYP2C8, ALDH3B2, GSTT1, GSTT2, CYP2E1, CYP1A2, ALDH3B1, MGST3, GSTP1, MGST1, MGST2 | 4.43E-38 |
| Drug metabolism | 43 | CYP3A4, CYP3A5, CYP3A7, CYP2C19, CYP2B6, CYP2C18, CYP2D6, ADH1C, ADH5, ADH6, ADH1B, ADH7, ADH1A, GSTM5, ALDH3A1, GSTM1, CYP3A43, GSTM2, CYP2A13, GSTM3, GSTM4, GSTK1, ALDH1A3, GSTZ1, GSTO2, GSTO1, GSTA1, GSTA2, GSTA3, GSTA4, GSTT2B, GSTA5, CYP2C9, CYP2C8, ALDH3B2, GSTT1, GSTT2, CYP2E1, CYP1A2, ALDH3B1, MGST3, CYP2A6, CYP2A7, GSTP1, MGST1, MGST2 | 4.02E-37 |
| Glutathione metabolism | 34 | GGT2, GGT1, GSTM5, GSTM1, GPX2, GSS, GSTM2, GPX1, GSR, GSTM3, GSTM4, GPX6, GPX5, GSTK1, GPX4, GPX3, GSTZ1, GSTO2, GPX7, GSTO1, GSTA1, GSTA2, GSTA3, GSTA4, GSTT2B, GSTA5, GSTT1, GSTT2, MGST3, GGT5, TXNDC12, GGT6, GGT7, GSTP1, MGST1, MGST2 | 5.24E-28 |
| Pyruvate metabolism | 29 | ME1, LDHC, LDHB, LDHA, ME3, ME2, LDHD, ACSS2, ALDH3A2, PDHB, ACSS1, ACOT12, ACYP2, ACYP1, PDHA2, GLO1, PDHA1, LDHAL6B, LDHAL6A, DLAT, HAGH, ALDH7A1, ALDH1B1, PKLR, DLD, ALDH2, HAGHL, ALDH9A1, PC | 1.58E-24 |
| Glycolysis / Gluconeogenesis | 34 | LDHC, LDHB, LDHA, ADH1C, HK2, ADH5, ADH6, ADH1B, HK1, ADH7, ADH1A, ACSS2, ALDH3A2, PDHB, ALDH3A1, G6PC2, ACSS1, HK3, ALDH1A3, PDHA2, PDHA1, LDHAL6B, LDHAL6A, ALDH3B2, DLAT, ALDH3B1, GPI, G6PC, ALDH7A1, GCK, ALDH1B1, PKLR, DLD, PGM1, ALDH2, ALDH9A1 | 2.90E-24 |
| Alanine, aspartate and glutamate metabolism | 24 | ADSS, ADSSL1, ASS1, ACY3, GLUD1, IL4I1, ASNS, CAD, AGXT, PPAT, GOT2, GLS2, ASPA, GAD2, GLUL, GOT1, GFPT1, GLS, GFPT2, ABAT, GPT, AGXT2, GAD1, GPT2 | 1.03E-20 |
| Cysteine and methionine metabolism | 20 | LDHC, LDHB, LDHA, LDHAL6B, MAT2A, LDHAL6A, IL4I1, TAT, GOT2, ADI1, CTH, GOT1, MAT1A, SDS, BHMT, MTR, MAT2B, AMD1, MPST, CBS | 2.44E-13 |
| Arginine and proline metabolism | 21 | ACY1, ASS1, GATM, GLUD1, ALDH3A2, CKB, GOT2, GLS2, ALDH7A1, CKMT1A, GLUL, CKM, GOT1, ALDH1B1, CKMT2, GLS, ALDH2, DAO, GAMT, AMD1, ALDH9A1 | 6.89E-10 |
| Valine, leucine and isoleucine biosynthesis | 11 | IARS, BCAT1, VARS2, BCAT2, LARS, PDHA2, PDHA1, IARS2, LARS2, VARS, PDHB | 1.72E-09 |
| Arachidonic acid metabolism | 21 | CYP2J2, CYP2C19, CYP2C18, CYP2C9, CYP2B6, CYP2C8, GGT2, GGT1, LTC4S, CYP2E1, GPX2, GPX1, GGT5, GGT6, GGT7, GPX6, PTGES, GPX5, GPX4, GPX3, GPX7, HPGDS | 2.32E-09 |
| Galactose metabolism | 15 | B4GALT1, LALBA, B4GALT2, HK2, HK1, G6PC2, G6PC, GCK, GLA, GANC, HK3, MGAM, PGM1, GAA, LCT | 5.89E-09 |
| Selenoamino acid metabolism | 14 | LCMT1, MAT2A, LCMT2, GGT2, GGT1, GGT5, GGT6, CTH, GGT7, SCLY, MAT1A, MARS2, MAT2B, MARS, CBS | 1.20E-07 |
| Glycine, serine and threonine metabolism | 15 | SHMT1, CHDH, SHMT2, GATM, PSPH, AGXT, CTH, SDS, DLD, BHMT, SRR, AGXT2, GAMT, DAO, CBS | 1.27E-07 |
| Propanoate metabolism | 15 | LDHC, LDHB, LDHA, LDHAL6B, LDHAL6A, ACSS2, ACSS3, ALDH3A2, ALDH7A1, ACSS1, ALDH1B1, ALDH2, ABAT, PCCB, ALDH9A1 | 2.16E-07 |
| Tyrosine metabolism | 17 | LCMT1, LCMT2, ADH5, ADH1C, ADH6, ALDH3B2, ADH1B, IL4I1, ADH1A, ADH7, COMT, TAT, ALDH3B1, ALDH3A1, GOT2, GOT1, ALDH1A3, TPO, GSTZ1 | 2.97E-07 |
| Histidine metabolism | 14 | LCMT1, ACY3, LCMT2, ALDH3B2, ALDH3A2, ALDH3B1, ALDH3A1, ALDH7A1, ASPA, ALDH1B1, ALDH1A3, HDC, ALDH2, ALDH9A1 | 6.78E-07 |
| Retinol metabolism | 18 | CYP3A4, CYP3A5, CYP3A7, CYP1A1, CYP2C19, CYP2C18, CYP2C9, CYP2B6, CYP2C8, ADH5, ADH1C, ADH6, ADH1B, ADH7, ADH1A, CYP1A2, CYP3A43, CYP2A13, CYP2A6, CYP2A7 | 1.09E-06 |
| Aminoacyl-tRNA biosynthesis | 15 | VARS2, DARS, SARS, AARS, QARS, DARS2, IARS2, LARS2, VARS, SARS2, IARS, LARS, MARS2, AARS2, MARS | 1.05E-05 |
| Starch and sucrose metabolism | 15 | SI, HK2, HK1, G6PC2, GBA3, GPI, G6PC, GCK, GANC, HK3, MGAM, PGM1, GAA, TREH, AGL | 1.50E-05 |
| Taurine and hypotaurine metabolism | 8 | GGT5, GGT6, GAD2, BAAT, GGT7, CSAD, GGT2, GGT1, GAD1 | 1.26E-04 |
| Cyanoamino acid metabolism | 7 | GBA3, GGT5, SHMT1, GGT6, SHMT2, GGT7, GGT2, GGT1 | 1.56E-04 |
| Purine metabolism | 26 | ADCY3, XDH, ADSS, ADCY4, ADCY1, ADCY2, ADCY7, ADCY8, NT5C1A, ADCY5, NT5C1B, ADCY6, PNP, ADA, PPAT, PFAS, NT5M, NT5C3, NT5C2, NT5E, NT5C, ADSSL1, GMPS, ADCY9, PKLR, PAICS | 3.39E-04 |
| Butanoate metabolism | 12 | ALDH7A1, GAD2, ALDH1B1, ALDH2, ABAT, PDHA2, PDHA1, GAD1, ALDH3A2, HMGCL, ALDH9A1, PDHB | 7.99E-04 |
| Linoleic acid metabolism | 11 | CYP3A43, CYP3A4, CYP3A5, CYP2J2, CYP3A7, CYP2C19, CYP2C18, CYP2C9, CYP2C8, CYP2E1, CYP1A2 | 9.15E-04 |
| Valine, leucine and isoleucine degradation | 13 | BCAT1, ACADSB, BCAT2, IL4I1, ALDH3A2, ALDH7A1, ALDH1B1, DLD, ALDH2, ABAT, PCCB, ALDH9A1, HMGCL | 0.001941902 |
| Tryptophan metabolism | 12 | KYNU, ALDH7A1, CYP1B1, CYP1A1, ALDH1B1, ALDH2, IL4I1, CYP1A2, ALDH3A2, INMT, ALDH9A1, AFMID | 0.004857796 |
| Phenylalanine metabolism | 9 | GOT2, GOT1, PRDX6, ALDH1A3, ALDH3B2, IL4I1, TAT, ALDH3B1, ALDH3A1 | 0.010351093 |
| Nicotinate and nicotinamide metabolism | 9 | NT5M, NT5C1A, NT5C3, NT5C1B, NT5C2, NADSYN1, NT5E, PNP, NT5C | 0.021694064 |
| Fatty acid metabolism | 11 | ACADSB, CHKB, ADH5, ADH1C, ADH1B, ADH6, ADH7, ADH1A, ALDH3A2, ALDH7A1, ALDH1B1, ALDH2, ALDH9A1 | 0.033555819 |
| Drug metabolism | 11 | CYP3A43, CYP3A4, XDH, CYP3A5, CYP2A13, CES2, CES1, CYP3A7, CYP2A6, CYP2A7, GMPS | 0.065891214 |
| beta-Alanine metabolism | 8 | ALDH7A1, GAD2, ALDH1B1, ALDH2, ABAT, GAD1, ALDH3A2, ALDH9A1 | 0.103063662 |
| Methane metabolism | 5 | SHMT1, MTHFR, SHMT2, PRDX6, ADH5 | 0.118240048 |
| One carbon pool by folate | 7 | MTHFD1, MTHFD2, SHMT1, MTHFR, SHMT2, MTR, MTHFD1L | 0.118521966 |
| Caffeine metabolism | 5 | XDH, CYP2A13, CYP2A6, CYP2A7, CYP1A2 | 0.264244274 |
| Amino sugar and nucleotide sugar metabolism | 10 | GPI, AMDHD2, GCK, GFPT1, HK3, PGM1, GFPT2, HK2, NPL, HK1 | 0.438071861 |
| Glycerophospholipid metabolism | 12 | CHKA, PLD2, PLD1, ACHE, CHKB, PHOSPHO1, PISD, PCYT1A, PCYT1B, PTDSS1, CHAT, PTDSS2 | 0.854106622 |
| Phenylalanine, tyrosine and tryptophan biosynthesis | 4 | GOT2, GOT1, IL4I1, TAT | 1.504234027 |
| Steroid hormone biosynthesis | 9 | CYP3A43, CYP3A4, CYP3A5, CYP17A1, CYP1B1, CYP3A7, CYP1A1, COMT, CYP19A1 | 2.753999077 |
| Limonene and pinene degradation | 5 | ALDH7A1, ALDH1B1, ALDH2, ALDH3A2, ALDH9A1 | 5.490001456 |
| Nitrogen metabolism | 6 | GLS2, CTH, GLUL, GLUD1, GLS, ASNS | 6.639916995 |
| Ether lipid metabolism | 7 | PLD2, PLD1, PAFAH2, PAFAH1B3, PLA2G7, PAFAH1B1, PAFAH1B2 | 9.828113418 |
| Ascorbate and aldarate metabolism | 5 | ALDH7A1, ALDH1B1, ALDH2, ALDH3A2, ALDH9A1 | 11.19827086 |
| D-Glutamine and D-glutamate metabolism | 3 | GLS2, GLUD1, GLS | 16.13168847 |
| Citrate cycle (TCA cycle) | 6 | DLD, PDHA2, PDHA1, DLAT, PDHB, PC | 21.95120083 |
| GnRH signaling pathway | 11 | ADCY3, ADCY4, PLD2, ADCY1, PLD1, ADCY2, ADCY7, ADCY9, ADCY8, ADCY5, ADCY6 | 31.57676068 |
| Glyoxylate and dicarboxylate metabolism | 4 | MTHFD1, MTHFD2, MTHFD1L, AFMID | 37.80749662 |
| Pyrimidine metabolism | 10 | NT5M, NT5C1A, NT5C3, NT5C1B, NT5C2, CAD, CTPS2, NT5E, PNP, NT5C | 51.53636189 |
| Progesterone-mediated oocyte maturation | 9 | ADCY3, ADCY4, ADCY1, ADCY2, ADCY7, ADCY9, ADCY8, ADCY5, ADCY6 | 62.25164194 |
| Glycerolipid metabolism | 6 | ALDH7A1, GLA, ALDH1B1, ALDH2, ALDH3A2, ALDH9A1 | 63.94007623 |
| Gap junction | 9 | ADCY3, ADCY4, ADCY1, ADCY2, ADCY7, ADCY9, ADCY8, ADCY5, ADCY6 | 68.14847101 |
